# Peri-tumor administration of 5-fluorouracil sol-gel using a hollow microneedle for treatment of gastric cancer

**DOI:** 10.1080/10717544.2018.1455760

**Published:** 2018-04-02

**Authors:** Yoon Suk Jung, Dong-Hoe Koo, Jeong-Yoon Yang, Hee-Young Lee, Jung-Hwan Park, Jung Ho Park

**Affiliations:** a Department of Internal Medicine, Kangbuk Samsung Hospital, Sungkyunkwan University College of Medicine, Seoul, South Korea;; b Department of Bionano Technology, Gachon University 65, Bokjeongdong, Gyeong Gi-Do, South Korea

**Keywords:** Peri-tumor administration, hollow microneedle, gastric cancer, 5-FU, sol-gel formulation

## Abstract

The aim of this study was to investigate the effectiveness of treating gastric cancer by injecting a pluronic F-127 sol-gel formulation of 5-fluorouracil (5-FU) into normal tissue surrounding the tumor using a hollow microneedle. The MTS tetrazolium assay was performed to assess the cytotoxicity of 5-FU after application to gastric cancer cells at different concentrations for 1, 5 and 10 h. Gastric cancer cells were inoculated subcutaneously into 30 male nude mice (CrjBALB/c-nu/nu mice, male); the inoculated mouse were divided into three groups. One group received no treatment, whereas the two other groups received free 5-FU gel (40 mg/kg) and 5-FU gel (40 mg/kg) for 4 days, respectively. Mean tumor volume, apoptotic index (TUNEL) and proliferative index (Ki 67) were evaluated in all groups. Cell viability was 77.3% when 1.22 g of free 5-FU was administered, whereas cell viability was 37.4% and 43.5% when 0.122 g of free 5-FU was administered per hour for 10 h and 0.244 g of free 5-FU was administered for 5 h (*p* < .01). The 5-FU sol-gel induced apoptosis and significantly inhibited cell proliferation compared to the free 5-FU (*p* < .01). In addition, xenografted tumor growth was significantly suppressed by administration of the 5-FU sol-gel formulation to inoculated mice (*p* < .01), and 71% (5/7) of xenografted tumors disappeared after 4 weeks. In conclusion, peri-tumor injection of a 5-FU sol-gel formulation into normal tissue surrounding the tumor mass using a hollow microneedle is an effective method for treating gastric cancer.

## Introduction

Gastric cancer is the third leading cause of cancer-related deaths in the world, and half of all worldwide cases occur in Eastern Asia (Ferlay et al., 2015). In Korea, a screening program was initiated in 1999 because early detection is associated with better clinical outcomes (Kong et al., 2004). However, advanced gastric cancer with gastric outlet obstruction (GOO) is found in a considerable number of patients, and the short-term prognosis of patients with GOO is generally very poor, with survival as short as 3–4 months (Jeurnink et al., 2007).

To relieve obstructive symptoms, two treatment modalities are generally used: palliative gastrojejunostomy (GJJ) and placement of self-expandable metal stents (SEMS). GJJ is associated with good functional outcomes and relief of symptoms in 72% of patients. However, it is associated with high morbidity (13%–55%) and mortality (2%–36%) (Jeurnink et al., 2010). Patients who have SEMS placed are more likely to tolerate earlier oral intake (average 7 days) and leave hospital earlier (average 12 days) with a comparable complication rate (average 15%–16%) (Ly et al., 2010), whereas more re-interventions caused by re-obstruction are required after SEMS placement when patients show good Eastern Cooperative Oncology Group (ECOG) performance or have an expected survival of 2 months or longer (Brimhall & Adler, 2011). Therefore, a new treatment method is needed to prevent re-obstruction in order to prolong survival and quality of life.

Polymer-based drug delivery systems have been investigated over the past few decades as local chemotherapy options for the treatment of tumors in cancer patients. Paclimer microparticles were investigated as a potential localized treatment for malignant glioma in the brain (Li et al., 2003) and recurrent ovarian cancer, but with limited success (Armstrong et al., 2006). Paclitaxel formulations of poly(ethylene glycol) tri-block copolymer (PLGA-PEG-PLGA), also known as ReGel and manufactured under the trade name OncoGel, were given intralesionally as an adjunct to radiotherapy in patients with inoperable esophageal cancer, and OncoGel plus radiotherapy reduced tumor burden as evidenced by dysphagia improvement, tumor size reduction, and negative esophageal biopsies (Aaron DuVall et al., 2009).

However, previous studies did not consider injection location, dose and method of administration, all of which had a large impact on tumor response. Direct administration of anti-cancer drugs into tumors has limitations because the drugs do not spread evenly throughout the tumor due to inflammation, necrosis and fibrosis present in the tumor. In addition, when cancer drugs are administered using conventional large-caliber injection needles, there is difficulty with needle penetration due to the mechanical strength of the tumor, and the relatively high interstitial pressure inside a tumor causes the drug to leak out and quickly disappear.

**Figure 1.**
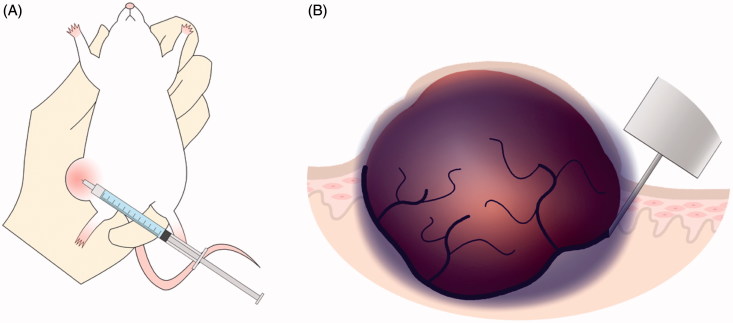
(A) Image of administering 5-FU around an artificially generated cancer tissue using a hollow micro needle. (B) A sustained 5-FU sol-gel formulation (purple area) was administered around the tumor to deliver the drug into the tumor.

Therefore, in this study, 5-fluorouracil, an anti-cancer drug, was formulated into a sustained-release formulation using a pluronic F-127 (PF-127). PF-127 exhibits unique thermoreversible gelling capacity. At low temperature in solution and as temperature is increased, the solubility of PF-127 is lowered, giving rise to the formation of a stable gel at 30 °C with sustained release of a drug (Unosson et al., 2016). This formulation was administered around the tumor to slowly release the drug and thereby affect the tumor over a long period of time. A hollow microneedle was used to administer the drug locally into the normal tissue around the tumor, and a sol-gel formulation was used to administer a sustained-released chemotherapeutic agent by syringe (Figure 1(A,B)). This study was conducted to investigate the effects of local administration of a sustained-release anti-cancer agent into normal tissues around the tumor.

## Materials and methods

### Materials

Pluronic F-127 (PF-127) and 5-fluorouracil (5-FU) were purchased from Sigma-Aldrich (St. Louis, MO).

### Preparation of 5-FU sol-gel formulation

F-127 (PF)-based formulations of 5-fluorouracil (5-FU) were prepared and the composition was as follows: PF-25/HPMC-1 [Pluronic 25% (w/w) and hydroxyl propyl methyl cellulose (HPMC) 1% (w/w)].

### Preparation of a hollow microneedle

NanoFil needles (31-gauge; Beckon-Dickinson, Rutherford, NJ) were wrapped in parafilm, leaving only 1.5 mm protruding from the parafilm. The hollow microneedle had a small inner diameter of 130 μm and an outer diameter of 205 μm.

### Release study of 5-FU gel

To establish differences in drug release between the general formulation and the sustained-release formulation, we used collagen gel. A cylinder hole with a diameter of 2 mm was formed in the center of each well of a 6-well culture plate. After injection of 100 μl of each of the fluorescein isothiocyanate (FITC) into the cylinder hole, plates were incubated and observed at 37 °C for 24 h. The drug release pattern was measured by photography. The diffusion rate of the two drug forms was quantified using Image J software.

### *In vitro* evaluation of cytotoxicity

#### Cell culture

Human gastric cancer cells (NCI-N87) were purchased from the Korean cell line bank and cultured in RPMI 1640 supplemented with L-glutamine (300 mg/L), 25 mM HEPES, 25 mM NaHCO_3_ and 10% heat-inactivated fetal bovine serum (FBS) at 37 °C in a 5% CO_2_ incubator.

#### Evaluation of cytotoxicity

The cytotoxicity of 5-FU against gastric cancer cells was determined using the CellTiter 96 AQueous One Solution Cell Proliferation assay (Promega Corp., Madison, WI,). NCI-N87 cells were seeded at a density of 5 × 10^3^ cells/well in a 96-well plate and incubated for 24 h. Cells were then incubated with RPMI medium containing 5-FU at various concentrations for 1 h, 5 h and 10 h after which media were replaced every hour with 100 μl RPMI medium containing 5-FU. After rinsing cells with PBS twice, 100 μl of fresh growth medium (RPMI) was added and cells were incubated for another 48 h. Media were then replaced with 100 μl of fresh growth medium followed by the addition of 20 μl of 3-(4,5-dimethylthiazol-2-yl)-5-(3-carboxymethoxyphenyl)-2-(4-sulfophenyl)-2 H tetrazolium (MTS) (Promega Corp.). After a further incubation period of 4 h, absorbance was measured at 490 nm using a BIO-RAD model 680 microplate reader. All experiments were carried out with four replicates.

### *In vivo* tumor suppression by 5-FU gel

#### Animals and tumor inoculation

Five-week-old male nude mice (Crj: BALB/c-nu/nu mice, male) were purchased from Orient Bio (Seongnam, Kyunggi-do, South Korea). All animal protocols were performed in accordance with the Animal Experiment Guidelines of Kangbuk Samsung Hospital and approved by the Animal Care Committee. To develop gastric cancer xenografts, mice were injected subcutaneously in the right back with a single-cell suspension containing 5 × 10^6^ cells/100 μl (PBS) under anesthesia. Mice were anesthetized by intraperitoneal injection of a ketamine-xylazine mixture (130 mg/kg ketamine and 8.8 mg/kg xylazine).

#### Administration of drugs with a hollow microneedle and *in vivo* tumor growth study

Drug injections were performed by using a hollow microneedle 4 weeks after the inoculation of gastric cancer cells (at a tumor size around 100–200 mm^3^). For local delivery of drugs into the tumor mass, free 5-FU and 5-FU sol-gel were prepared and injected into the subcutaneous layer of normal tissue right next to the tumor mass at a single dose of 40 mg/kg 5-FU. The 5-FU sol-gel was stored at 4 °C to prevent the change from sol to gel. Preliminary experiments were performed to determine an effective drug injection schedule; this led to the establishment of the following injection schedule: 2-day infusion of 5-FU, 2-day rest, and 2-day infusion of 5-FU. Experimental animals were divided into three groups: PF-127, free 5-FU and 5-FU sol-gel. Injections were administered according to the schedule, and changes in lesion size were observed every week for one month.

### Tumor development evaluation

#### Caliper measurements

Tumor volume was calculated using the mean diameter measured with Vernier calipers and the following formula: volume = 0.5 × *a* × *b*^2^, where *a* and *b* are the smallest and largest diameters, respectively. A complete response (CR) was recorded if the mass had disappeared at 4 weeks, whereas a partial response (PR) was recorded if the size of the mass had decreased by more than 25% after 4 weeks. All other cases were recorded as no response (NR).

#### Apoptosis and proliferation index assay (Jones et al., 1997)

All nude mice were euthanized using CO_2_ gas one month after drug injection. Tumor tissues were divided in half; one half was frozen in liquid nitrogen, while the other was fixed in 10% neutral formalin for 24 h, after which paraffin blocks were prepared. For the apoptosis assay, nicked DNA ends were labeled by the terminal deoxynucleotidyl transferase-mediated cUDP nick end labeling (TUNEL) using the *In Situ* Cell Death Detection Kit (Roche, Basel, Switzerland) following the manufacturer's protocol. As a final step, tissue sections were counterstained with methylgreen (Sigma-Aldrich). Apoptotic cells identified by the TUNEL assay were quantitated under brightfield microscopy. The apoptotic index was determined by enumerating the number of positively labeled cells per crypt epithelial cell units in at least three well-oriented crypt epithelial cell units (Hall et al., 1994). This was expressed as the mean number multiplied by a factor of 100. A proliferation index (PI) was determined by enumerating the number of cells that stained with anti-human Ki 67 rabbit monoclonal antibody (Immunoteck, Marseille, France) in at least three well-oriented crypt epithelial units, and expressed as the mean percentage of the total number of cells counted in each crypt epithelial unit.

### Statistical methods

Two-tailed Student's *t*-tests and ANOVA tests were utilized to assess the statistical significance of differences among groups. A *p*-value of <.05 was considered to be significant. All data are expressed as means ± standard deviations.

## Results

### Release characteristics of 5-FU gel formation

**Figure 2.**
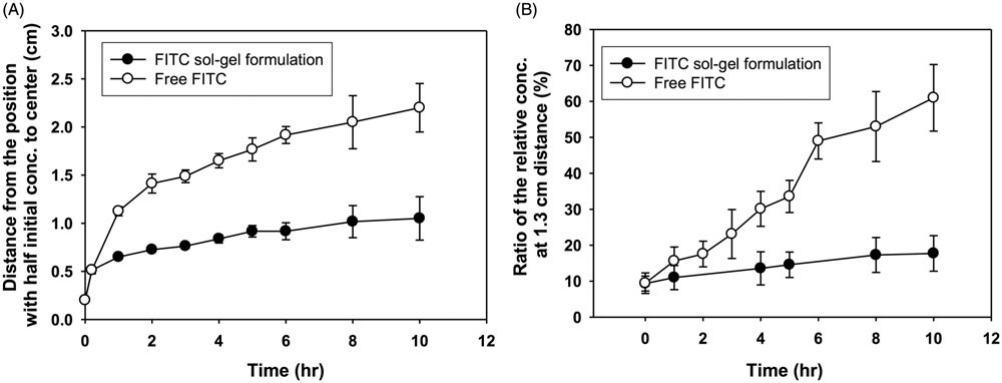
Drug release profiles of free FITC and FITC sol-gel formulation after injection in collagen gel. (A) Diameters at the half maximum concentration of the drug. (B) The ratio of the relative concentration value (measured concentration/initial concentration) at 1.3 cm of the distance from the center over time.

*In vitro* release behaviors of free FITC and FITC sol-gel formulation are shown in Figure 2. Free FITC diffused faster than the 5-FU gel formulation, and the diameter of the region corresponding to half of the initial concentration of drug increased rapidly, as shown in Figure 2(A). When the local concentration of FITC at each site was compared with the initial drug concentration, they were equivalent to 56%, 20%, 10% and 6%, respectively. The concentration of FITC from free FITC and FITC gel at 1.3 cm away from the center was compared with the initial concentration of FITC (Figure 2(B)). In the case of free FITC, the concentration of FITC at a distance of 1.3 cm from the center of the administration site was 15% of the initial concentration at 1 h after administration. In the case of the FITC sol-gel formulation, the concentration of the FITC increased slowly at each site 1.3 cm from the center of the administration site over time. At 5 h, the concentration of free FITC at 1.3 cm distance was twice as high as that of FITC gel at the same distance. There was a significant difference in the maximum cumulative release rate at each position in the two groups (*p* < .01) (Figure 2(A,B)), with the free FITC diffusing more than twice as fast as the FITC sol-gel formulation.

### *In vitro* growth inhibition study

**Figure 3.**
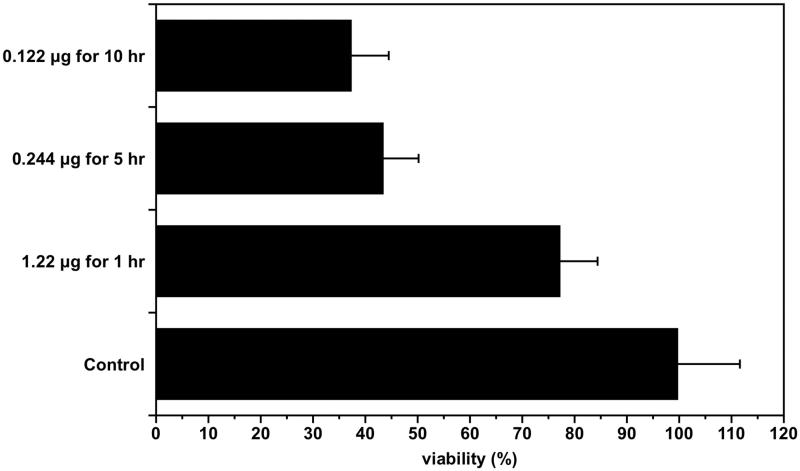
Assessment of the cytotoxic effects regarding administration methods of Free 5-FU. The effect of the total dose of the free 5-FU at once was compared with the effect of the total dose of free 5-FU administered in multiple times to see the effect of the sustained release.

The purpose of this experiment was to compare the anti-cancer effect according to the administration method even though the total dose of the anti-cancer drug was the same. The effect of the total dose of the anti-cancer drug administered all at once was compared with the effect of the total dose of the anti-cancer drug administered multiple times to see the effect of the sustained release. Cells were treated with 1.22 μg of free 5-FU once for 1 h, 0.244 μg five times for 5 h and 0.122 μg 10 times for 10 h. The MTS method was used to measure the cancer cell death rate as a result of each treatment. There was no significant difference in the degree of cancer cell death between the group treated with 0.244 μg five times for 5 hr and the group treated with 0.122 μg 10 times for 10 h, but cell viability by treatment with 1.22 μg of 5-FU once for 1 h was greater than the other two groups (*p* < .01) (Figure 3).

### *In vivo* anti-tumor activity: size measurement using calipers

**Figure 4.**
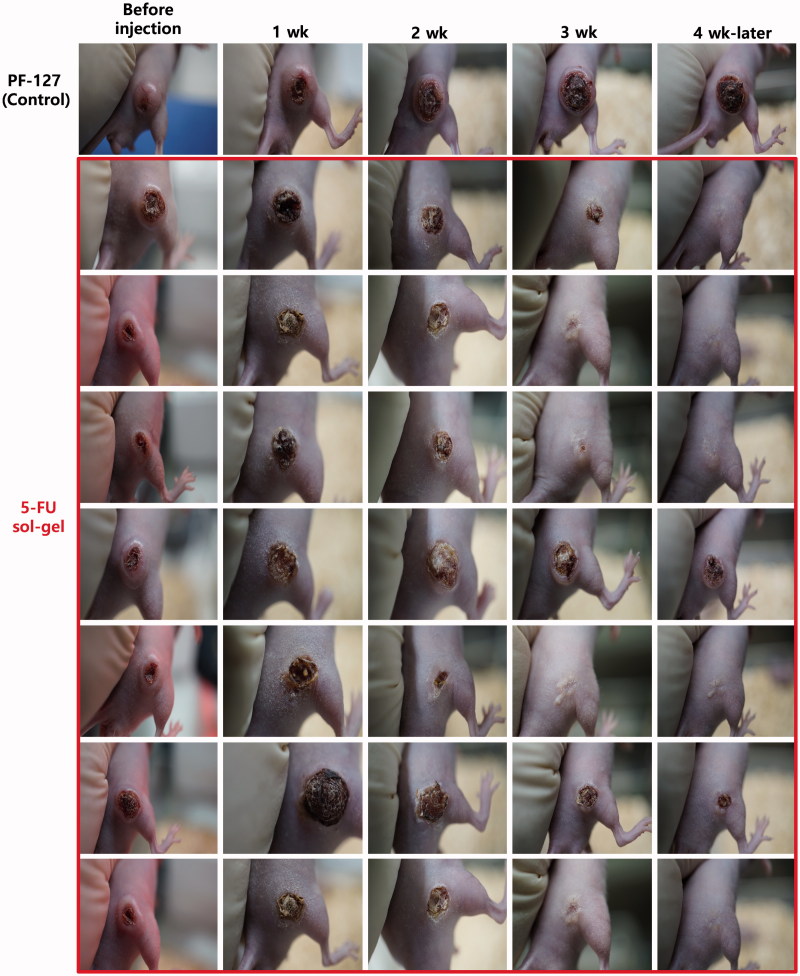
Changes in tumor size of gastric carcinoma after administration of gel only w/o 5-FU and 5-FU gel. Human gastric cancer cells were xenografted into 8 rats to evaluate the efficacy of 5-FU gel.

**Figure 5.**
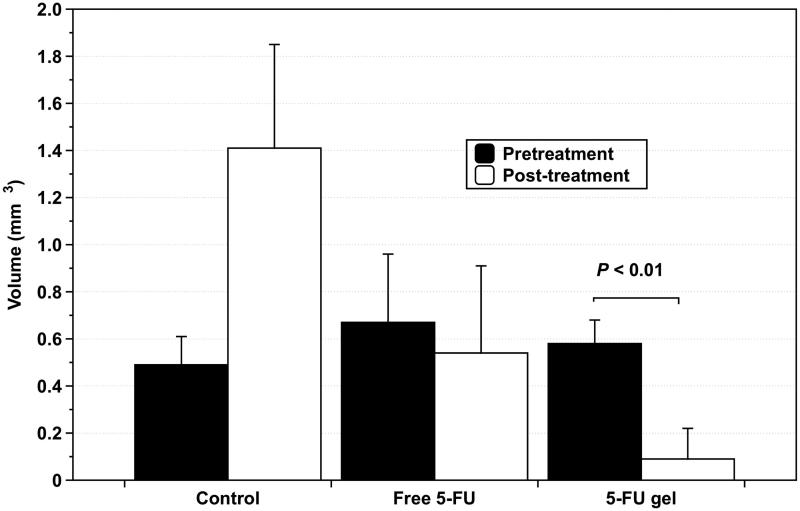
Comparison of mean tumor volume by peri-tumor administration of gel only w/o 5-FU, free 5-FU, and 5-FU gel.

Animal models of human gastric cancer cells xenografted into rats were used to evaluate the efficacy of 5-FU gel. Treatment efficacy of 5-FU gel was compared to that of PBS (control) and free 5-FU. Drugs were administered according to the schedule described previously: 2-day infusion of 5-FU, 2-day rest and 2-day infusion of 5-FU. Both free 5-FU and 5-FU gel were able to inhibit tumor growth, whereas the group treated with gel only without 5-FU showed a high tumor growth rate. Local injection of 0.52 mg of free 5-FU resulted in a CR in one mouse (1/8) and a decrease in size of the mass (PR) in one mouse (1/8), with no response (NR) observed in the other six mice (6/8). In contrast, mice treated with 5-FU gel showed a higher anti-tumor response (Figure 4). Local injection of 5-FU gel (1.04 mg) resulted in a CR in 5/7 mice and a significant decrease in the size of the mass (PR) in one mouse (1/7), with no response in one mouse (1/7; Figure 4). By comparing the mean volumes of the tumor masses, it was confirmed that the inhibition of tumor growth by treatment with 5-FU gel was significantly more effective than with free 5-FU treatment, as shown in Figure 5 (*p* < .01).

### Apoptosis and proliferation index (PI) assay

**Figure 6.**
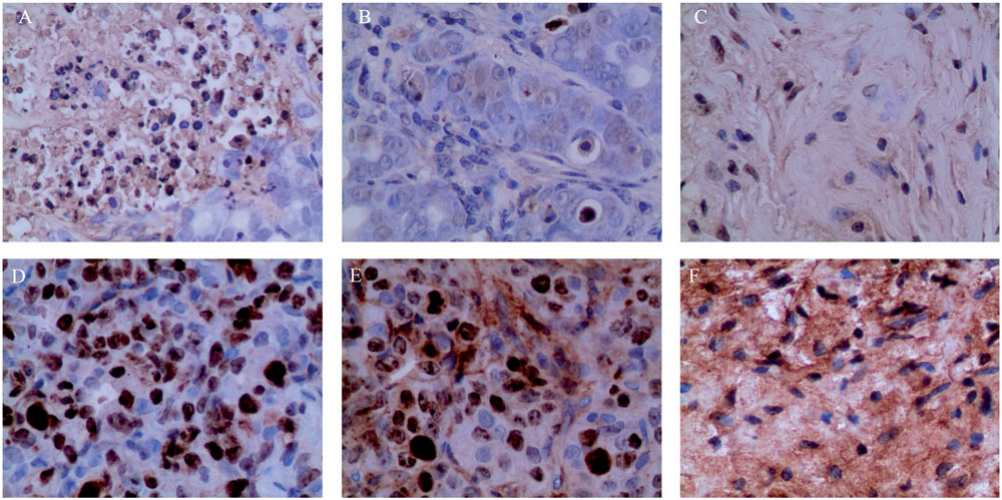
Apoptosis and proliferation index of the (A & D) control, (B & E) partial response, and (C & F) complete response groups.

The apoptotic index in the control and partial response groups was below 0.1% (Figure 6(A,B)). In the complete response group, cancer cells were not observed, and the apoptotic index was 0 (Figure 6(C)).

When measuring the ratio of positive cells to the Ki 67 antibody used as the cell proliferation index, the percentage of positive cells was 41.2% for the control group, 28% for the partial response group, and 0% for the complete response group, respectively, as shown in Figure 6 (D–F).

## Discussion

In this study, injection of a sustained-release formulation of 5-FU gel into the normal tissue around the tumor using a hollow microneedle was effective in bringing about tumor cell death. The 5-FU sol-gel formulation was a solution at room temperature that turned into a gel at body temperature and gradually released the drug. In particular, *in vitro* experiments demonstrated that maintaining a locally high concentration of 5-FU in normal tissues around the tumor for a relatively long time was effective for treatment. Gastric cancer is basically an adenocarcinoma arising from the transformation of epithelial cells. Because gastric cancer is mainly in the epidermis, a hollow microneedle was used to administer drugs effectively near the dermal layer. In addition, the hollow microneedle can administer the drug near the dermis by integrating it with the endoscope. Because the efficacy of tumor cell killing by locally injected 5-FU is proportional to the local concentration of 5-FU, the administration of 5-FU several times was more effective than the administration of a larger amount of drug at one time. These results are consistent with previous findings (Calabro-Jones et al., 1982; Meta-analysis Group In Cancer et al., 1998).

According to our *in vivo* experimental results, the majority of tumors in the group treated with 5-FU sol-gel disappeared within 4 weeks after treatment. These results indicate that local injection into tissue surrounding the tumor (peri-tumor injection) is more effective than intra-tumoral injection of anti-cancer drugs (Nakase, 2004). These results are due to the following reasons. First, by injecting the anti-cancer drug locally into the surrounding normal tissue of the tumor rather than directly into the tumor mass, the drug was able to spread around large areas of the tumor. Although direct injection of a drug into the tumor results in a rapid local tumor response, (Hohenforst-Schmidt et al., 2013) the rapid loss of the drug from the tumor occurred due to high interstitial fluid pressure, and the distribution of the drug is likely to be uneven due to the abnormal vasculature of the tumor and structural abnormalities of the tumor center (Minchinton & Tannock, 2006; Hohenforst-Schmidt et al., 2013). Second, when the drug was administered using a hollow microneedle, the highly concentrated formulation was injected locally at the desired site without leaking. Third, drug administration into the subcutaneous or submucosal layer by a hollow microneedle showed a delayed *t*_max_ (time that a drug is present at the maximum concentration) compared to intramuscular and subcutaneous administration (Jun et al., 2015). Finally, use of a hollow microneedle was a more effective method than a standard size needle for local administration of a highly concentrated drug to a narrow therapeutic region.

Pluronic F-127 (PF-127) is a synthetic hydrogel made of amphiphilic copolymers consisting of units of ethylene oxide (PEO) and polypropylene oxide (PPO), and is widely used in drug delivery and controlled release because of its biocompatibility (Ruel-Gariépy & Leroux, 2004). In a previous study using other hydrogels, release of drugs from 2-hydroxyethylmethacrylate- and acrylic acid-based 5-FU imprinted hydrogels was sustained for only 5 h (Chung et al., 2009). For PF-127, the duration of the drug release depends on the concentration of PF-127. When 25% PF-127 was used, phenylephrine released locally over 10 h. In this study, a formulation with 25% PF-125 and 1% HPMC was used. Duration of 5-FU for 10 h can affect cancer cells in various cell cycles. However, long-lasting drug release is not always desirable because drug administration may have to be stopped depending on the patient's condition. In addition, the long-term exposure of anti-cancer drugs to the tissues may lead to an increased incidence of cancer (Witt & Bishop, 1996; Glen & Dubrova, 2012). Also, in the case of the sustained-release formulation, which was required to release the drug for a long period of time, the initial dose was too large to deliver a large amount at a time.

Initially, the 5-FU sol-gel formulation was administered daily for 4 days. This administration schedule in initial animal tests was the same as intravenous administration of 5-FU to patients with gastric cancer. However, this administration schedule was changed to administering for 2 days, resting for 2 days, and administering again for 2 days. The reason for the change in the administration schedule was that toxic side effects were observed after administration to the rats and the half-life of 5-FU was about one day. No side effects from drug toxicity were found after changing the administration schedule. In this study, 1 mg of 5-FU sol-gel was administered to 1 cc of tumor volume. Considering these doses, the administration schedule used in this study can be used for patients with gastric cancer.

When 5-FU was exposed at different concentrations for various times of 1, 5 or 10 h but the total amount was the same. The survival rates of gastric cancer cells were evaluated to determine whether the 5-FU sol-gel formulation was more effective than the free drug. When 5-FU was administered orally, the concentration in the cancer tissue was about 0.122 μg/g (Fukunaga et al., 1987). The drug concentration of 5-FU administered for 1 hr was determined to be 1.22 μg in the tumor. After a single intravenous infusion of 5-FU, 5-FU disappeared from plasma rapidly and no measurable 5-FU was detected after 2 h (Fraile et al., 1980). Given these findings, the exposure time of the drug to cancer cells was set at 1 h. The death of cancer cells was significantly greater when a single dose of 5-FU was divided into several doses for 5 h or 10 h rather than one time. The degree of cancer cell death was influenced by the concentration of 5-FU, but the duration of the effective drug concentration was more important than the dose when a sufficient concentration of 5-FU was administered. Local administration of a sufficient amount of a sustained-released formulation of 5-FU increased treatment effectiveness and reduced side effects compared to systemic 5-FU treatment by intravenous injection.

Inhibition of drug diffusion by sol-gel formulation was observed through *in vitro* experiments. A collagen medium has been used as a model for diffusing drugs into tissues because this medium has diffusion properties similar to those of tissue (Cheema et al., 2012). At 1.3 cm from the center of the administration site, the FITC concentration was about 10% of the initial FITC concentration. These results indicated that the FITC sol-gel formulation could maintain an effective drug concentration around the tumor for a longer period of time than the free FITC. FITC with a molecular weight of 389 g/mol was slightly larger than 5-FU with a molecular weight of 130 g/mol. Therefore, the diffusion rate of 5-FU will not be significantly different from the diffusion rate predicted by FITC (Modok et al., 2007). These results showed that multiple administrations of the sustained-release formulation maintained the therapeutic concentration for a long time around the tumor.

## Conclusion

Injection of a sustained-release 5-FU sol-gel formulation using a hollow microneedle effectively induced the death of gastric cancer cells. In addition, local administration of a slow-release 5-FU formulation around a tumor may help to resolve local problems in the GI tract, such as gastric outlet obstructions. Thus, this treatment can be considered when surgery is difficult due to the poor condition of the patient and/or when systemic chemotherapy or radiotherapy are no longer viable options.
